# Iowa

**Published:** 1896-02

**Authors:** 


### IOWA.

An Act to Repeal Chapter 50 of the Twenty-fourth General Assembly 1892, and provide a substitute therefor, and to enlarge the duties and powers of the State Dairy Commissioners and provide an appropriation therefore.

*Be it Enacted by the General Assembly of the State of Iowa:*

Section 1. That chapter 50 of the Twenty-fourth General Assembly of 1892 is hereby repealed, and the following enacted in lieu thereof:

If any person or corporation shall sell or exchange or expose for sale or exchange, deliver or bring to another for domestic use or to be converted into any product of human food whatsoever, any unclean, impure, unhealthy, adulterated or unwholesome mik, or milk from which has been held back, what is commonly known as strippings, or milk taken from an animal having disease, sickness, ulcers, abscesses, or any running sore, or was taken from an animal less than fifteen days before or less than five days after parturition, or should sell or offer for sale any diseased or unwholesome meats, or where the flesh of one animal has been substituted for that of another; or for from animals in advanced stages of pregnancy, or which have recently given birth to young, shall upon conviction thereof be fined not less than $25.00 nor more than $100.00, and be liable in double the amount of damages to the person or persons upon which such fraud shall be committed; provided that the provisions of this act shall not apply to skimmed milk or condemned meats when sold as such.

Sec. 2. For the purposes of this act, milk which is proved by any reliable method or test or analysis, to contain less than three pounds of butter fat to the one hundred pounds of milk, shall be regarded as skimmed milk, or cows more than eight months and sows and ewes more than fourteen weeks pregnant, shall be considered as advanced in pregnancy, and those that have given birth to young less than ten days before time of slaughter, shall be considered as having recently given birth to young.

Sec. 3. It is hereby made the duty of the State Dairy Commissioner to enforce the provisions of the foregoing sections.

Sec. 4. The State Dairy Commissioner shall, as soon after this act goes into effect as convenient, and biannually thereafter on March 1st, appoint in each city within the State, having 5000 or more inhabitants, a regularly graduated veterinarian, who shall act as local dairy and meat inspector for a period of two years, or until his successor has been lawfully appointed.

Sec. 5. It shall be the duty of said inspectors to personally inspect each dairy at least once a month, and examine into the sanitary condition of the barns, milk rooms and utensils, food, and water supply, and make a critical examination of the health of each cow in dairy, and make the necessary milk tests; he shall also personally, and at frequent intervals, inspect and examine into the sanitary condition of each meat market, stall, shop, store-room, warehouse and their contents, in or about which any milk, meats, fish, oysters, birds or fowls are kept, held, or offered for sale as human food.

Sec. 6. It shall be the duty of said inspectors upon discovering any unsanitary condition of the barns, milk-room, meat market, stall, shop, warehouses, or storehouse, utensils, or vehicles, used to store, draw, or deliver any milks, meats, fish, oysters, birds, or fowls, to order the same to be put into a sanitary condition within twenty-four hours, or as soon thereafter as practical, or should he discover any diseased, adulterated, unwholesome, or impure milk, or any diseased or unwholesome meat, fish, oysters, birds, or fowls, he shall at once notify the person or persons in whose possession said articles are found, to at once remove the same to such place as said inspector shall designate for its destruction as human food, or should any inspector find any cow or cows at a dairy so diseased as to affect the milk, thereby rendering it unwholesome, he shall at once quarantine said animal or animals, as far as the use of the milk is concerned, and in case of tuberculosis, or should the inspector suspect tuberculosis, he shall at once notify the State Dairy Commissioner, who may at his discretion order the inspector to test the herd with tuberculin, the necessary material and assistance to be furnished by the commissioner, and any cattle found to be diseased with tuberculosis shall be disposed of according to the laws of the State governing said disease.

Sec. 7. Local inspectors shall receive as full compensation for their services salaries as follows: In cities having from 5000 to 10,000 inhabitants, $480 per annum; in cities having from 10,000 to 15,000 inhabitants, $600 per annum; in cities having from 15,000 to 25,000 inhabitants, $900 per annum; in cities having from 25,000 to 50,000 inhabitants, $1200 per annum; in cities having from 50,000 to 75,000 inhabitants, $1500 per annum; payable monthly; and before entering upon the duties of his office each inspector shall, in addition to the oath of office or affirmation required by law, execute a bond to the State of Iowa in the penal sum of $1000 for every $500, or fraction thereof, he shall receive in salary.

Sec. 8. Each person, firm, or corporation conducting a dairy, milk depot, or meat market or stall, shall procure a license from the State Dairy Commissioner, through the local inspector, and shall pay such license fee as hereinafter prescribed. A dairy license fee shall be 50 cents per head for each cow used in said dairy, milk depot; wholesaling, $10.00, and for retailing, $5.00. Each meat market wholesaling or jobbing meats shall pay a license fee of $50.00, and each retailer of meats shall pay a license fee of $25.00.

Each license shall be annual, and terminate on March 31 of each year.

All license fees collected by the local inspectors shall be turned over to the State Dairy Commissioner, and by him to the State Treasurer.

Sec. 9. Any person or persons who shall violate, disobey, omit, neglect, or refuse to comply with, or who resists any of the provisions of this act, or who omits, neglects, or refuses to comply with the orders of said inspector, or who resists said inspectors in carrying out any of the requirements of this act, shall upon conviction thereof be fined not less than $25.00 nor more than $200.00, or be imprisoned not less than thirty nor more than one hundred and twenty days, or both, at the discretion of the Court.

Any person or firm convicted of violating any of the provisions of this act shall, in addition to the prescribed fine, forfeit their license fee for the current term.

Sec. 10. The State Dairy Commissioner is hereby authorized to make rules and regulations for the enforcement and carrying out the purposes and intents of this act.

Sec. 11. There is hereby appropriated out of any of the State funds not otherwise appropriated, the sum of $ for the carrying out of this act for the next biennial period, provided not more than one-half of the same shall be used before March 31, 1897.

